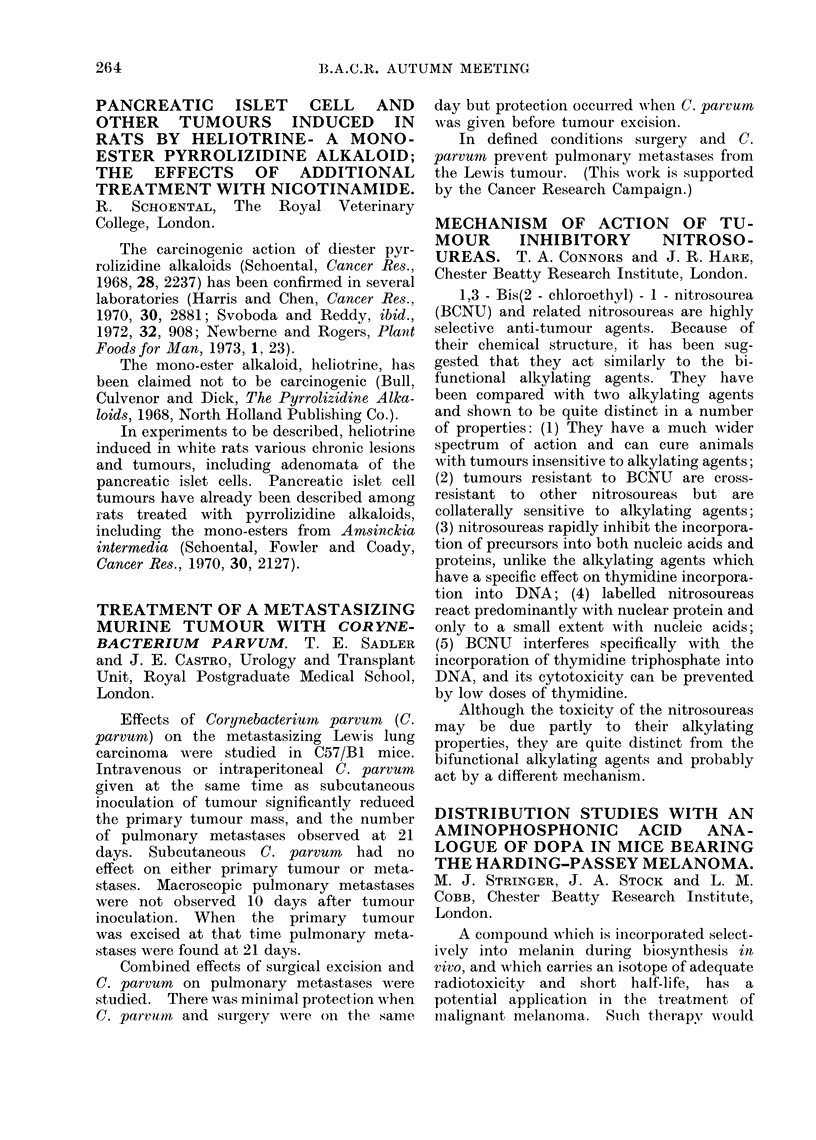# Proceedings: Mechanism of action of tumour inhibitory nitrosoureas.

**DOI:** 10.1038/bjc.1975.51

**Published:** 1975-02

**Authors:** T. A. Connors, J. R. Hare


**MECHANISM OF ACTION OF TUMOUR INHIBITORY NITROSOUREAS.** T. A. CONNORS and J. R. HARE, Chester Beatty Research Institute, London.

1,3 - Bis(2 - chloroethyl) - 1 - nitrosourea (BCNU) and related nitrosoureas are highly selective anti-tumour agents. Because of their chemical structure, it has been suggested that they act similarly to the bifunctional alkylating agents. They have been compared with two alkylating agents and shown to be quite distinct in a number of properties: (1) They have a much wider spectrum of action and can cure animals with tumours insensitive to alkylating agents; (2) tumours resistant to BCNU are cross-resistant to other nitrosoureas but are collaterally sensitive to alkylating agents; (3) nitrosoureas rapidly inhibit the incorporation of precursors into both nucleic acids and proteins, unlike the alkylating agents which have a specific effect on thymidine incorporation into DNA; (4) labelled nitrosoureas react predominantly with nuclear protein and only to a small extent with nucleic acids; (5) BCNU interferes specifically with the incorporation of thymidine triphosphate into DNA, and its cytotoxicity can be prevented by low doses of thymidine.

Although the toxicity of the nitrosoureas may be due partly to their alkylating properties, they are quite distinct from the bifunctional alkylating agents and probably act by a different mechanism.